# Mitochondrial mutations drive prostate cancer aggression

**DOI:** 10.1038/s41467-017-00377-y

**Published:** 2017-09-22

**Authors:** Julia F. Hopkins, Veronica Y. Sabelnykova, Joachim Weischenfeldt, Ronald Simon, Jennifer A. Aguiar, Rached Alkallas, Lawrence E. Heisler, Junyan Zhang, John D. Watson, Melvin L. K. Chua, Michael Fraser, Francesco Favero, Chris Lawerenz, Christoph Plass, Guido Sauter, John D. McPherson, Theodorus van der Kwast, Jan Korbel, Thorsten Schlomm, Robert G. Bristow, Paul C. Boutros

**Affiliations:** 1 Informatics and Biocomputing Program, Ontario Institute for Cancer Research, Toronto, ON Canada M5G 0A3; 2 Genome Biology Unit, European Molecular Biology Laboratory, Heidelberg, 69120 Germany; 3 Biotech Research & Innovation Centre (BRIC) and Finsen Laboratory, Copenhagen, 2200 Denmark; 4 Institute of Pathology, University Medical Center Hamburg-Eppendorf, Hamburg, 20246 Germany; 5 Princess Margaret Cancer Centre, University Health Network, Toronto, ON Canada M5G 1L7; 6 Division of Theoretical Bioinformatics, German Cancer Research Center, Heidelberg, 69120 Germany; 7 Division of Epigenomics and Cancer Risk Factors, German Cancer Research Center, Heidelberg, 69120 Germany; 8 Genome Technologies Program, Ontario Institute for Cancer Research, Toronto, ON Canada M5G 0A3; 9 Department of Pathology and Laboratory Medicine, Toronto General Hospital/University Health Network, Toronto, ON Canada M5G 2C4; 10 Martini-Clinic, Prostate Cancer Center, University Medical Center Hamburg-Eppendorf, Hamburg, 20246 Germany; 11 Department of Medical Biophysics, University of Toronto, Toronto, ON Canada M5G 1L7; 12 Department of Radiation Oncology, University of Toronto, Toronto, ON Canada M5T 1P5; 13 Department of Pharmacology and Toxicology, University of Toronto, Toronto, ON Canada M5S 1A8

## Abstract

Nuclear mutations are well known to drive tumor incidence, aggression and response to therapy. By contrast, the frequency and roles of mutations in the maternally inherited mitochondrial genome are poorly understood. Here we sequence the mitochondrial genomes of 384 localized prostate cancer patients, and identify a median of one mitochondrial single-nucleotide variant (mtSNV) per patient. Some of these mtSNVs occur in recurrent mutational hotspots and associate with aggressive disease. Younger patients have fewer mtSNVs than those who diagnosed at an older age. We demonstrate strong links between mitochondrial and nuclear mutational profiles, with co-occurrence between specific mutations. For example, certain control region mtSNVs co-occur with gain of the MYC oncogene, and these mutations are jointly associated with patient survival. These data demonstrate frequent mitochondrial mutation in prostate cancer, and suggest interplay between nuclear and mitochondrial mutational profiles in prostate cancer.

## Abstract (web summary)

In prostate cancer, the role of mutations in the maternally-inherited mitochondrial genome are not well known. Here, the authors demonstrate frequent, age-dependent mitochondrial mutation in prostate cancer. Strong links between mitochondrial and nuclear mutational profiles are associated with clinical aggressivity.

## Introduction

Prostate cancer remains the most prevalent non-skin cancer in men^1^ and exhibits a remarkably quiet mutational profile^2^. Exome-sequencing studies of localized tumors have revealed few recurrent somatic single-nucleotide variants (SNVs)^3, 4^, while whole-genome sequencing studies have not identified highly recurrent driver non-coding SNVs or genomic rearrangements (GRs)^5–8^. Although strong mutagenic field effects have been observed^9, 10^, their underlying mechanisms and to what extent they drive tumor initiation or progression are unknown. Nevertheless, promising molecular diagnostics predictive of aggressive disease have been created using supervised machine-learning techniques, both from RNA abundance data^11, 12^ and from DNA copy number data^13^, showing strong linkage between molecular features of prostate tumor cells and patient outcome.

Most studies of the prostate cancer genome have focused on mutations occurring in the nuclear genome, and have ignored the other genome of the cell: the mitochondrial genome. Mitochondria are maternally inherited and play critical roles in pathways dysregulated in cancer cells, including energy production, metabolism and apoptosis^14^. It is therefore critical to evaluate the status of the mitochondrial genome to have a complete view of the overall mutational profile of prostate cancer. While mitochondrial mutations have been observed in several tumor types^15–17^, including prostate cancer^18–22^, their global frequency and clinical impact have not yet been comprehensively characterized. Previous studies have found that mitochondrial mutations are associated with increased serum prostate-specific antigen (PSA) levels^21^, have suggested that mtDNA mutations increase cancer cell tumorigenicity^20^, and indicate that overall mitochondrial mutation burden is correlated with higher Gleason Scores^22^.

To characterize the mitochondrial mutation landscape of prostate cancer, we analyzed the mitochondrial genomes of 384 adenocarcinomas of the prostate across all National Comprehensive Cancer Network (NCCN) defined risk categories, including 164 early-onset prostate cancers (EOPCs, age at diagnosis less than 50). We identify recurrent mutational hotspots in the mitochondrial genome, which included recurrently mutated bases or recurrently mutated genes or regions. We also confirm increasing mutation burden with patient age^23–26^, identify interactions between nuclear and mitochondrial mutation profiles and reveal specific mitochondrial mutations enriched in aggressive prostate tumors.

## Results

### Mitochondrial genome sequence analysis

We collected 384 tumors from patients with localized prostate cancer, comprising 164 EOPCs and 220 late-onset prostate cancers (LOPC; Supplementary Data 1; Supplementary Fig. 1). The LOPC patients represented the three NCCN risk groups: 19 low-risk, 151 intermediate-risk, and 36 high-risk. The average sequencing depth of the mitochondrial genome was 13,577×, allowing extremely sensitive mutation detection. This cohort does not include any nuclear whole-genome duplication events, as demonstrated by SNP microarray analysis^7^. We first evaluated the mitochondrial copy number (MCN) for each sample from the sequencing coverage of the mitochondrial and nuclear genomes. MCN ranged from 75 to 1405 (mean: 431) across the cohort, and was strongly associated with age (linear model, *P* = 1.67 × 10^−26^), as well with clinical indices such as T-category (ANOVA, *P* = 6.01 × 10^−3^) and Gleason Score (GS; ANOVA, *P* = 6.46 × 10^−3^; Supplementary Fig. 2).

**Fig. 1:**
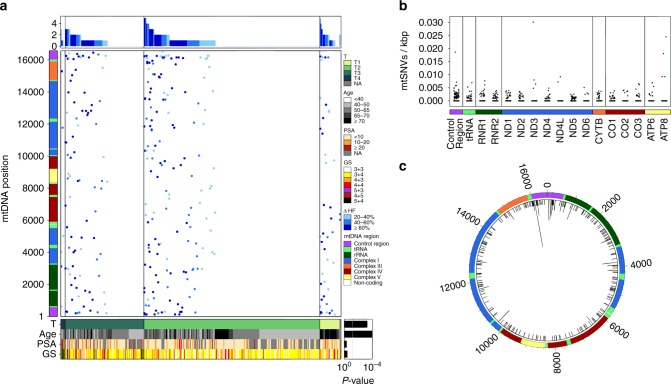
Panorama of mitochondrial mutations in prostate cancer. **a** The *top panel* displays the number of mtSNVs per patient sorted first by T-Category and then by the number of mtSNVs; histogram bars are colored by the average difference in the heteroplasmy fraction (∆*HF*) between tumor and normal samples, *light-blue* 20–40%, *medium-blue* 40–60%, *dark-blue* ≥60%. A heatmap showing the location of each mtSNV on the mitochondrial genome (*middle*), where the color of each dot represents ∆HF. The mitochondrial genome is represented on the *left*. The *bottom panel* shows the clinical covariates for all 384 patients: Age, Gleason Score, PSA, and T-Category. *Bottom right*: Associations between the covariates and number of mtSNVs. **b** Frequency and distribution of single-nucleotide variants (*SNVs*) within the mitochondrial genome. Mutation frequency normalized by dividing the number of mutations per locus of each patient by (length of the locus (kbp) × MCN). **c** Distribution of mtSNVs across the mitochondrial genome. mtSNVs were fairly evenly distributed across the genome (*black bars*) and recurrent mutation positions are indicated by the histogram

We next conservatively identified mitochondrial SNVs (mtSNVs) as those positions that had an absolute difference in their heteroplasmy fraction (∆HF) between purity-adjusted tumor and paired-normal samples of at least 0.20 (Supplementary Fig. 1). Because the number of identified mtSNVs is dependent on the heteroplasmy fraction threshold, we chose to balance false positives and false negatives with an intermediate value. There were 293 mtSNVs across all patients, with 47.4% of tumors (182 out of 384) harboring at least one and 6.8% (26 out of 384) harboring three or more (see “Methods” section, Fig. 1a; Supplementary Data 2). Proportions of patients with 0, 1, 2, ≥3 mtSNVs are 202 out of 384 (52.6%), 110 out of 384 (28.6%), 46 put of 384 (12.0%), and 26 out of 384 (6.8%), respectively. The number of patients with no mtSNVs was greater than expected by chance, suggesting significant variability in mtSNV burden (permutation test; *P* = 3.4 × 10^−5^). Tumors with a larger number of mitochondria were more likely to have an mtSNV (generalized linear model (GLM) family binomial; *P* = 8.38 × 10^−7^). mtSNVs were associated with tumor size (T-category; *X*^2^ test; *P* = 2.47 × 10^−4^), but not other clinical prognostic indices like pre-treatment PSA and GS (Fig. 1a). PCR followed by Sanger sequencing validated 18 out of 25 predicted mtSNVs (Supplementary Fig. 3; Supplementary Fig. 4; Supplementary Table 1), suggesting precision of ~ 75%, comparable to somatic indel detection accuracy^27^.

### Frequently mutated mitochondrial loci

The noncoding control region of the mitochondria (mtDNA positions: 1–576 and 16,024–16,569), was the most frequently mutated region with 15.4% (59 out of 384) of tumors harboring mutations in that region (Supplementary Data 2; Supplementary Fig. 5). The control region comprises several elements, including the heavy- and light-strand promoters, as well as the origins of replication for the heavy strand (OHR), two hypervariable regions (HV1, HV2) and three conserved sequence blocks (CSB1, CSB2, CSB3). All functional locations were defined from mitmap.org^28^. Of these regions, HV1 was the most frequently mutated (mtDNA positions: 16,024–16,383). Overall, mutation rates were generally consistent across regions of the mitochondrial genome (Fig. 1b).

There were 157 mtSNVs in the 13 protein coding genes, 82% (129 out of 157) of which were nonsynonymous, including six premature stop codons and two mutated stop codons. The most frequently mutated protein-coding gene was *ND5* (30 out of 157). We identified 21 specific positions mutated in at least two patients (Fig. 1c): 10 within the control region, eight in protein-coding regions and three in rRNA subunits. Of the coding mutations, seven were non-synonymous and one introduced a premature stop codon. In the control region, position 16,093—a common site of tissue specific heteroplasmy^29, 30^—was the most frequently mutated position (nine patients; Fig. 1c). Of protein-coding genes, *ND1* was frequently mutated, with two patients having G3946A mutations (∆HF: 0.63, 0.24), leading to a structure-disrupting E214K amino acid change, resulting in a reduction of complex assembly^31^. A second mutation, G4142A was found in two patients (∆HF: 1.0, 0.21; R279Q) and a third mutation, G3842A, in three patients (∆HF: 0.45, 0.21, 0.95; premature stop codon).

There were 22 mutations within mitochondrial tRNA genes, and eight of these were located within anticodon stems. In *CO1*, there were non-synonymous mutations at G5910A (A2T in one patient; ∆HF: 0.84), and T6664C (I254T in one patient; ∆HF: 0.46), two amino acids previously observed to be mutated in prostate cancer cells^20^. Two patients with mutations at position 6419 were detected within the *CO1* gene (∆HF: 0.2, 0.23), although these two showed heteroplasmy within the normal samples and homoplasmy in the tumor, suggesting that these mtSNVs represent either tissue-specific heteroplasmy^32^ or mutations that have gone to fixation in the tumor. Overall, *CO1* was mutated in 4.7% (18 out of 384) of patients, markedly lower than the 11% rate previously reported^20^.

### Age effect on the distribution of mtSNVs in prostate cancer

**Fig. 2:**
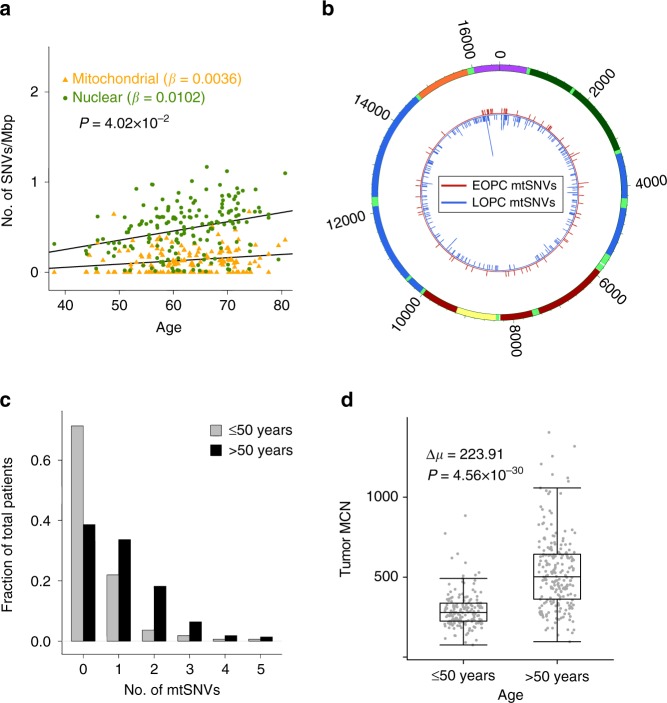
The difference in mitochondrial mutational frequency and copy number with age. **a** Association of nuclear (*green*) and mitochondrial (*yellow*) mutation SNV/Mbp rates with patient age. Mitochondrial mutation rate normalized by MCN. **b** Distribution of mtSNVs in EOPC (*red*) and LOPC (*blue*) patients. The histogram indicates presence and frequency of a mtSNV. The most recurrent mtSNV was at position 16093. **c** The fraction of patients by number of mtSNVs, EOPC (*gray bars*), LOPC (*black bars*). **d** Tumor mitochondrial copy number (*MCN*) for both patient age groups. EOPC: *n* = 164; LOPC: *n* = 220

As expected, the occurrence of mitochondrial mutations was strongly associated with patient age (GLM family binomial; *P* = 5.88 × 10^−9^; Fig. 1a)^23–26^. The mitochondrial mutation rate was significantly lower than that of the nuclear genome mutation rate (Fig. 2a; *P* = 0.040, F-test), which may in part be explained by differential mutation detection accuracy in the two genomes. To further understand the association of mtSNVs with age, we separated patients into those 50 and under years of age (EOPC; *n* = 164) and those over 50 (LOPC; *n* = 220). The median ages of the EOPC and LOPC cohorts were 47 and 63.5 years old, respectively. Patients with EOPC were significantly more likely to have no mitochondrial mutations, 117 out of 164 (71.3%), than those with LOPC (85 out of 220, 38.6%; *P* = 4.22 × 10^−10^, proportion test; Fig. 2b, c). Despite this difference in mutational load, the two groups have similar distributions of mtSNVs across the mitochondrial genome, with the highest fraction of mtSNVs within the control region (Supplementary Fig. 6). EOPC patients had about 224 fewer copies of the mitochondria than LOPC patients (Mann–Whitney test; *P* = 4.56 × 10^−30^; Fig. 2d). This effect was inverted in the normal samples with EOPC patients having 86 more copies (Mann–Whitney; *P* = 1.54 × 10^−14^; Supplementary Fig. 2d), consistent with the decline in lymphocyte MCN with age^33^.

### Associations between mtSNVs and nuclear genomic mutations

**Fig. 3:**
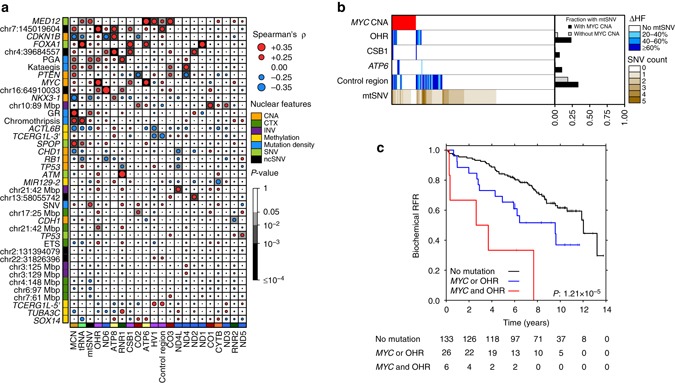
Associations between mitochondrial and nuclear genome mutations. **a** Correlations of mitochondrial features with nuclear genome features. The size and color of the dot represents the Spearman correlation and the background shading represents the *P*-value. Nuclear features: SNVs, CTXs, INVs, kataegis data available for 172 patients; Chromothripsis: *n* = 159; CNAs: MYC, NKX3-1 (*n* = 203); CDH1, CDKN1B, CHD1, PTEN, RB1, TP53 (*n* = 194); Methylation: *n* = 104. Mitochondrial features: 216 patients. **b** Mutations in OHR are associated with CNAs in MYC. Heatmap showing those patients with CNA gains (*red*) in MYC and those with mtSNVs in OHR, CSB1, the control region and ATP6, mtSNV color represents the ∆HF. Since CSB1 is a subregion within OHR, mutations in CSB1 are also considered as OHR mtSNVs, similarly, mtSNVs in OHR are also within the control region (*n* = 203). The *bar plot* on the *right* shows the fraction of patients with or without a *MYC* CNA that have a specific mtSNV. **c** Kaplan–Meier plot of 165 patients with OHR and *MYC* mutations. Patients were grouped according to whether they had neither *MYC* CNAs nor OHR SNVs (*black line*), a *MYC* CNA or an OHR mtSNV (*blue*) or had both (*red line*). The group that had a CNA gain in *MYC* and an mtSNV in the OHR region had significantly worse outcomes than those without the mutations. Biochemical *RFR* Biochemical relapse-free rate

Intriguingly, mutations in the large rRNA subunit (*RNR2*) were significantly correlated with mutations in the mitochondrial gene *ND4* (Spearman’s *Ρ* = 0.19; *P* = 0.00015), suggesting to us an inter-play between different mutational types. To rigorously assess this phenomenon, we studied mutational associations between the nuclear and mitochondrial genomes. We exploited a set of 40 candidate nuclear somatic driver events recently identified through recurrence analyses, including five measures of mutation density, six methylation events, six non-coding SNVs, five coding SNVs, five measures of mutational density, ten genomic rearrangements and eight copy number aberrations (CNAs)^7^. The SNVs included recurrent coding SNVs in genes that are commonly mutated in prostate cancer, as well as the six most recurrent non-coding SNVs. To characterize per-region mtSNVs, we defined 22 mutational features representing the broad functional aspects of the mitochondria, 13 protein coding genes, 2 rRNAs, tRNAs (treated as one group), the control region and 3 subregions within the control region, along with mtSNV number and MCN. For each of the nuclear features, we evaluated their correlation to 22 mitochondrial mutational features in 194 LOPCs with the nuclear mutational data (Supplementary Data 3). We detected multiple nuclear-mitochondrial mutational associations (Fig. 3a). For example, SNVs in *FOXA1* were significantly positively correlated with multiple mitochondrial features, as were SNVs in *MED12*. Nuclear-mitochondrial correlations were weakly dependent on the ∆HF threshold used to call mtSNVs (Supplementary Fig. 7, Supplementary Data 4).

One prominent nuclear-mitochondrial mutational interactions was co-occurrence of *MYC* copy number gain and mtSNVs within the OHR (Fig. 3b). Mutations within the OHR may dysregulate mtDNA replication, while *MYC* induces mitochondrial biogenesis by activating genes required for mitochondrial function^34^ and influences metabolic plasticity in cancer stem cells^35^. Risk of biochemical failure (BCR) after primary definitive treatment by radiotherapy or surgery was significantly higher for patients whose tumors harbored both *MYC* CNAs and OHR mtSNVs relative to those with neither or one of these two mutations, suggesting a synergistic mitochondrial-nuclear effect on disease aggression (Fig. 3c). Several other similar instances of apparent synergistic mitochondrial-nuclear effects on disease aggression were observed (Supplementary Fig. 8a–e), suggesting that this is a common phenomenon in prostate cancer. While we have used the region defined as OHR (mtDNA positions: 110–441) as the mitochondrial feature, this subregion of the Control Region significantly overlaps with a region defined as HV2 (mtDNA positions: 57–372). We confirmed that HV2 mtSNVs show the same synergistic effect with *MYC* CNAs as mtSNVs defined as OHR (Supplementary Fig. 8f). Interestingly, *MYC* CNAs were more common in LOPCs (14.5%; 29/200) than in EOPCs (8.4%; 10/119) making it impossible to assess if the same nuclear-mitochondrial interactions occur in both disease states. Further evaluation of changes in nuclear-mitochondrial associations across disease progression will be revealing.

### Clinical impact of mtSNVs in prostate cancer

**Fig. 4:**
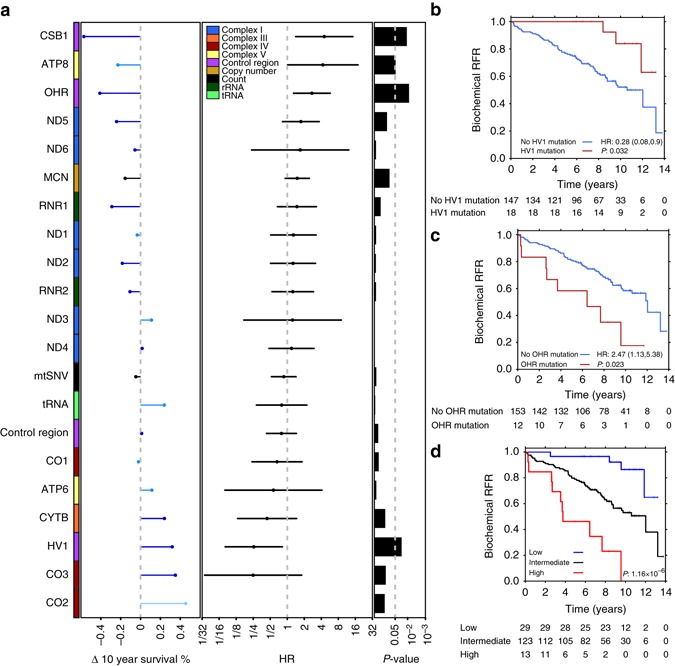
Clinical impact of mitochondrial mutations in prostate cancer. **a** The associations of biochemical recurrence (BCR) and 21 mitochondrial features: 19 mitochondrial genes or regions, MCN (median-dichotomized), and mtSNV count (0 vs. 1 + ) were calculated using Cox models in 165 LOPC patients. Hazard ratios (HRs) are shown in the *middle panel* and *P*-values from the log-rank test in the *right panel*. The change in the 10-year survival for patients with mutations in each mitochondrial region is indicated (*left panel*). The color of the bars indicate the average ∆HF for mtSNVs in that region; *light-blue* 20–40%, *medium-blue* 40–60%, *dark-blue* ≥60%. **b** Kaplan–Meier plots of mtSNVs occurring within HV1 and (**c**) OHR. **d** Kaplan–Meier plot of results of leave-one-out cross-validation predictions (*P*-value from log-rank test)

The recurrence of mitochondrial mutations in specific regulatory regions and their association with prognostic nuclear mutations strongly suggested their ability to drive disease aggression. We therefore systematically evaluated the association of individual mitochondrial somatic mutational features with disease aggression in 165 patients with clinical follow-up using Cox proportional hazards modeling. Of our 22 mitochondrial mutational features (Fig. 3a), four were significantly associated with biochemical relapse rates (Fig. 4a; Supplementary Table 2): mutations in CSB1, OHR, *ATP8* and HV1. We should note that MT-ND4L was not included in this analysis as only one patient of the 165 had a mtSNV in this gene. To evaluate if these mutations were independent prognostic variables, we employed multivariable modeling to adjust for age, pre-treatment PSA, T-category and GS. After adjustment, mtSNVs in HV1 were associated with better patient outcome (Fig. 4b; Hazard Ratio, HR = 0.28, 95% CI = 0.08–0.9, *P* = 0.032, Wald test), while mtSNVs in OHR were associated with significantly worse patient outcome (Fig. 4c; HR = 2.47, 95% CI = 1.13–5.38, *P* = 0.023, Wald test).

These data suggested that mtSNVs might comprise a novel way to predict patient outcome. We therefore assessed the ability of a multi-mtSNV signature to identify patients at elevated risk for biochemical failure (who therefore might benefit from treatment intensification) and those at low risk (who might therefore be appropriate for surveillance protocols). Using leave-one-out cross-validation and univariate feature-selection, we created a three-class signature that separated patients into three distinct risk groups for biochemical failure (Supplementary Fig. 9a). The signature identified both patients at elevated risk (Fig. 4d; HR = 3.41, 95% CI = 1.71–6.80, *P* = 0.0005, Wald test) and patients at low-risk (HR = 0.23, 95% CI = 0.08–0.65, *P* = 0.005, Wald test). These effects are independent of clinical features: when we considered only the clinically-homogeneous NCCN intermediate risk group, the same mtSNV signature again separated three groups with distinct risk profiles (Supplementary Fig. 10). The cross-validation method identified seven genes (*CO2*, *CO3*, *ATP8*, HV1, OHR, CSB1, *ND4L*) as informative for classification. Patients with mtSNVs in (*CO2*, *CO3*, HV1) were classified as low-risk and patients with mtSNVs in (*ATP8*, OHR, *ND4L*, CSB1) were classified as high-risk. To show that this does not lead to over-fitting, we chose the three most frequently mutated regions of the seven (*CO3*, HV1, OHR), which also clearly separated patients into three groups (Supplementary Fig. 9b).

## Discussion

The mitochondrial mutational landscape of cancer has been relatively unexplored. Previous work has shown a large-scale mtDNA deletion has predictive value in the prostate biopsy outcomes^36^, suggesting the feasibility of mtDNA-based molecular tests. We identify a large number of mtSNVs in localized prostate cancer. These mutations show complex interplay with nuclear mutational characteristics, and appear to work together to drive tumor aggressiveness.

Mitochondrial mutations also show associations with risk of biochemical relapse. Interestingly, mtSNVs within the control region can have conflicting outcomes; however, when separated into the different noncoding subregions (HV1, OHR), we found that certain loci were associated with better outcomes and others with worse outcomes. The overlap of the OHR and HV2 within the control region and their association with *MYC* CNAs highlight the need for better understanding of the functions of the control region^37^. In future, treating the control region as distinct regulatory regions may provide further insight into the roles of these regions, as well as any contribution they may make toward tumor aggression. We note that the number of pairs of nuclear-mitochondrial mutational features tested may elevate false-positive rates, and it will be key to perform validation studies in larger cohorts to verify their effect-sizes and biological significance.

The differences observed in the mitochondrial mutational profiles of EOPC and LOPC patients show a need to better understand the association between mtSNVs and aging, and how this may relate to the development of prostate cancer. While the MCN of matched-normal samples decreases with patient age, a previously observed trend^33^, tumor MCN estimates were significantly higher in older patients, which could account for the higher frequency of mtSNVs in these patients. However, since the majority of the samples of each age group come from different research centres, this striking difference in tumor MCN will require further investigation to exclude any confounding effects.

Further studies will be needed to assess when different mtSNVs occur during tumor evolution and their timing relative to common nuclear mutations. The function of many of these mtSNVs is unclear, and functional and mechanistic studies linking them to tumor evolution and mitochondrial function will be of great interest. This will more clearly identify the mitochondrial mutations that are important for mitochondrial-nuclear communication and how they may interact to drive tumor formation. Localized prostate cancer remains the most diagnosed non-skin cancer in men, and identification of aggressive disease remains an urgent clinical dilemma. Addition of mtSNVs to prognostic biomarkers may be an effective way of improving prediction of patient outcome, supporting triage of patients with low-risk disease to surveillance protocols and with high-risk disease to adjuvant therapy regimens.

## Methods

### Patient cohort

We collected 384 prostate cancer tumor samples with matched normal samples (381 blood, 3 tissue-derived). The samples had Gleason Scores ranging from 3 + 3 to 5 + 4. The 165 patients from the Canadian Prostate Cancer Genome Network (CPC-GENE) underwent either radical prostatectomy or image-guided radiotherapy as detailed in Fraser et al.^7^. In addition, 51 samples from publicly available data sets were included in the somatic mutation analysis and correlations with clinical variables, age, Gleason Score and T-category^4–6, 8^, three of TCGA samples had tissue-derived normal samples as opposed to blood-normals. All samples were manually macro-dissected and were assessed by an expert urological pathologist to have tumor cellularity >70%. All tumor specimens were taken from the index lesion. Publicly available tumor tissues were obtained and used following University Health Network Research Ethics Board (REB) approved study protocols (UHN 06-0822-CE, UHN 11-0024-CE, CHUQ 2012-913:H12-03-192). Local REB and ICGC guidelines were used to collect whole blood and informed consent from CPC-GENE patients at the time of clinical follow-up.

### EOPC patient cohort and sample processing

We collected 168 tumor samples from EOPC patients. Informed consent and an ethical vote (institutional reviewing board) were obtained according to the current ICGC guidelines. The patients did not receive any neo-adjuvant radiotherapy, androgen deprivation therapy, or chemotherapy prior to the surgical removal of tumor tissue. Tumor samples and a normal blood control were frozen at −20 °C and subsequently stored at −80 °C.

### EOPC DNA library preparation, sequencing and alignment

DNA library preparation and whole-genome sequencing was performed on Illumina sequencers with the raw length of the reads displaying a median of 101 bp. Reads were aligned to the hg19 reference genome using BWA-MEM version 0.7.8-r455 [arXiv:1303.3997v2] and duplicates were removed using Picard (http://broadinstitute.github.io/picard). Mitochondrial reads were extracted using SAMtools^38^.

### Nuclear mutation calling

Recurrent nuclear genomic features were obtained from Fraser et al.^7^, which included five recurrent coding SNVs from commonly mutated genes in prostate cancer; the six most recurrent noncoding SNVs; CNAs from eight commonly mutated prostate cancer genes; the 10 GRs included the five most recurrent translocations and the four most recurrent inversions plus a recurrent inversion containing the PTEN gene; the TMPRSS-ERG fusion; presence or absence of kataegis events; chromothripsis; three metrics of mutation density (median dichotomized PGA estimates, number of SNVs and number of GRs); six methylation events were identified through univariate CoxPH modelling as associated with disease progression. Nuclear somatic SNVs were predicted by SomaticSniper (v1.0.2)^39^, (*n* = 172 samples), setting the mapping quality threshold to 1, otherwise with default parameters. Nuclear SNVs were filtered using SAMtools (v0.1.6)^38^ and SomaticSniper (v1.0.2) provided filters, as well as a mapping quality filter and false-positive filter from bam-readcount (downloaded 10 January 2014). Nuclear SNVs were then annotated by ANNOVAR (v2015-06-17)^40^. The nuclear mutation rate was obtained by dividing the number of SNVs after filtering by the number of callable loci. CNAs were analyzed by Affymetrix OncoScan microarrays (*n* = 194) and the methylation data were generated by Illumina Infinium Human Methylation 450k BeadChip kits (*n* = 104). Genomic rearrangements were called using Delly (v0.5.5)^41^ (*n* = 172). Chromothripsis scores (*n* = 159) were calculated by ShatterProof (v0.14)^42^ and subsequently dichotomized with a 0.517 threshold. Sample processing, whole-genome sequencing and whole-genome sequencing data analysis are as described in detail by Fraser et al.^7^.

### Mitochondrial SNV calling

Reads mapped to the mitochondria during whole-genome alignment were extracted using BAMQL (v1.1)^43^ using the command:   bamql -I -o out_mito_reads.bam -f input_wgs.bam '(chr(M) & mate_chr(M)) | (chr(Y) & after(59000000) & mate_chr(M))';

The second part of the query statement collects reads, where one of the pair mapped to chrM and the other unmapped, which in our data was also assigned to an unresolved region in chrY.

The output files from BAMQL were used as input bam files for the mitochondrial genome analysis program MToolBox (v0.2.2)^44^. The versions of the various system requirements were: Python v2.7.2; gmap v.2013-07-20^45^; samtools v0.1.18^38^; java v1.7.0_72; picard v1.92 (http://broadinstitute.github.io/picard); muscle v3.8.31^46^. We used default parameters for MToolBox and used the default RSRS^47^ as the reference genome. The default parameters include a minimum base quality score of 25, samples that failed the MToolBox program using default parameters, but successfully completed at a lower base quality parameter setting of 20, were nonetheless removed from the analysis.    MToolBox_v0.2.2/MToolBox.sh -i bam -r RSRS -M -I -m '-D genome_index/ -H hg19RSRS -M chrRSRS' -a '-r genome_fasta/ -F -P -C'

The predicted mitochondrial genome for each tumor sample and the number of reads supporting each base were compared to the corresponding normal sample, if available, from each patient. Positions where the absolute difference in heteroplasmy fraction (∆HF) was >0.2 were considered to be mitochondrial SNVs (mtSNVs). While this does not preclude the possibility of tissue-specific heteroplasmy being mislabeled as somatic mutations, this allowed us to identify somatic variants as well as ignore those positions that could be called population variants, reducing the number of potentially false positive variant calls. Heteroplasmy fraction estimates were adjusted to account for tumor cellularity using cellularity values calculated by qpure^48^. Tumor HF values were adjusted with the following equation:

$${\rm Tumor}\,{\rm{H}}{{\rm{F}}_{{\rm{cellularity}}}} = ( {{\rm{Tumor}}\,{\rm{H}}{{\rm{F}}_{{\rm{MToolBox}}}} - ( {1 - {\rm{cellularity}}} )}\\ {*\,{\rm{Normal}}\,{\rm{H}}{{\rm{F}}_{{\rm{MToolBox}}}}} )/{\rm{cellularity}}\hskip3pc$$

If there were no cellularity values available, we assumed cellularity = 1.0. Those values of Tumor HF_cellularity_ that were less than zero or greater than one were rounded to zero and one, respectively.

In the mitochondrial reference genome, there are three positions encoded as “N” to preserve historical numbering, (523, 524 and 3107), in addition position 310 is located within a homopolymer region and is a common variant^28^. These four positions can result in misalignments^49^, therefore they were filtered out of our analyses, as in previous studies^50^. We also filtered out those positions with relatively low coverage of <100 read depth. Positions of mitochondrial genes and subregions of the noncoding control region were obtained from http://www.mitomap.org. Pathogenicity scores from MutPred^51^, PolyPhen-2^52^ and SiteVar^53^ were obtained from the MToolBox output. Mutations in tRNA genes were compared to the Mamit-tRNA database^54^.

We chose to a threshold of 0.2 ∆HF in order to balance removing false positives without excluding a large number of mtSNVs unnecessarily (Supplementary Fig. 7c). As part of this assessment, we looked at four correlations between different nuclear and mitochondrial features using mtSNVs assessed at increasing ∆HF cutoffs from 0.1–0.6 (Supplementary Fig. 7, Supplementary Data 4). In each of these four cases, raising ∆HF from 0.1 to 0.2 led to increasing correlation coefficients between the two features. Three of the correlations that were not significant at 0.1 ∆HF became significant at higher ∆HF, suggesting that some mtSNVs with lower HF values may be either false positives or low-level tissue-specific heteroplasmies. Any further increases in ∆HF had differing effects on the four correlations.

### mtDNA copy number

Mitochondrial copy number per cell was calculated using the equation: (mitochondrial coverage/nuclear coverage) ×2, using nuclear coverage data from the whole-genome alignment^7^ and mitochondrial coverage data calculated by bedtools genomecov (v2.24.0)^55^. The mitochondrial mutation rate per megabase DNA was calculated by dividing the number of mtSNVs by the tumor MCN multiplied by the number of callable bases, 16565, accounting for the 4 positions that were removed.

### Survival and statistical analyses

The mtSNV data were compared to patient clinical features in the R statistical environment (v3.2.3). Binomial regression (age, PSA) and Chi-square tests (T-category, Gleason Score) were used to identify associations between the clinical variables and mtSNVs for all 384 patients. Survival analyses were performed on 165 patients due to survival data availability. Cox proportional hazards models were used to calculate HRs for mtSNVs in the different mitochondrial features such as genes or MCN, with verification of the proportional hazards assumption. The mitochondrial feature MT-ND4L was removed from this analysis as only one patient in the 165 cohort had a mtSNV in this gene. Change in 10 year percent survival was calculated using survival rates. Kaplan–Meier plots were created comparing biochemical recurrence with the presence or absence of mutations in certain mitochondrial loci, (genes or noncoding regions) or median-dichotomized tumor MCN. Nuclear genomic features were chosen based on recurrence in a previous prostate cancer study^7^. The data were visualized using the R-environment and lattice (v0.20-31), latticeExtra (v0.6-26) and circos (v0.67-4)^56^. Associations between nuclear and mitochondrial genome features were calculated using Spearman’s correlation.

### PCR validation

Single-nucleotide variants in mitochondrial DNA were validated by Sanger re-sequencing, as previously reported^7^. Briefly, 10 ng of total genomic DNA (including mitochondrial DNA) was subjected to PCR amplification using primer pairs flanking SNVs identified from whole-genome sequencing (Supplementary Table 3). The sequence data surrounding the region of interest were obtained from http://www.mitomap.org/bin/view.pl/MITOMAP/HumanMitoSeq. The amplicon sequence generated by the in silico PCR was then entered into the NCBI genome BLAST search engine to identify non-mitochondrial sequences that were similar. This was done to ensure that there were some differences between the designed primers and nuclear sequences, as well as to identify any sequence regions that could confound downstream analyses. The genome used for the BLAST search was GRCh38.p2 reference assembly top-level. These web pages were used on 20 and 21August 2015 and verified on 13 September 2016. PCR reactions were purified using the QIAquick PCR purification kit (Qiagen, Toronto, Canada). Sanger re-sequencing was performed using amplicon-specific primers on an ABI 3730XL capillary electrophoresis instrument (Thermo Fisher Scientific, Burlington, Canada) at The Centre for Applied Genomics, Hospital for Sick Children, Toronto, Canada.

### Data availability

The sequencing data are available at the European Genome-Phenome Archive (EGA) repository under accession EGAS00001001782.

## Electronic supplementary material

Supplementary Data 1

Supplementary Data 2

Supplementary Data 3

Supplementary Data 4

Supplementary Information
