# Supplementary material for: Mitochondrial mutations drive prostate cancer aggression
Source: Nat Commun. 2017 Sep 22;8:656. doi: 10.1038/s41467-017-00377-y (PMC5610241; doi:10.1038/s41467-017-00377-y)
Supplement: Supplementary file 5 — Supplementary Information [file 41467_2017_377_MOESM5_ESM.pdf]

File name: Supplementary Information
Description: Supplementary Figures and Supplementary Tables

File name: Supplementary Data 1
Description: Clinical and sequencing data per patient The data includes patient age at treatment, Gleason Score, T-category, PSA (ng/mL) level, tumour cellularity, number of mtSNVs and the mean coverage depth, mitochondrial copy number for both normal and tumour sample and the aligner used for each wgs. The presence or absence of mutations in each of 20 mitochondrial regions and MYC and NKX3-1 copy number aberrations is indicated for each sample and the amount of DNA that was sent for sequencing for the CPC-GENE samples are included.

File name: Supplementary Data 2
Description: Table of 293 somatic mtSNVs List of mtSNVs, including heteroplasmic fractions (HF), reference allele nucleotide, identity of tumour and normal major alleles and major allele heteroplasmy fractions (both adjusted and unadjusted by tumour cellularity), tumour and normal coverage at each position, the mtDNA gene or region and pathogenicity scores from MutPred and Polyphen2 obtained from MToolBox.

File name: Supplementary Data 3
Description: Mitochondrial mutation recurrence for 41 nuclear genomic features The table includes the number of patients that had a specific nuclear genome CNA, GR, methylation event or SNV and of those patients the number that also harbours an mtSNV in any of 22 mtDNA features.

File name: Supplementary Data 4
Description: Table of mtSNVs with ΔHF values between 0.1 and 0.2 List of 265 mtSNVs, that had ΔHF values greater than 0.1, but less than 0.2. The table includes heteroplasmic frequencies, reference allele nucleotide, identity of tumour and normal major alleles and major allele heteroplasmy fractions, tumour and normal coverage at each position and the mtDNA gene or region.

Supplementary Information

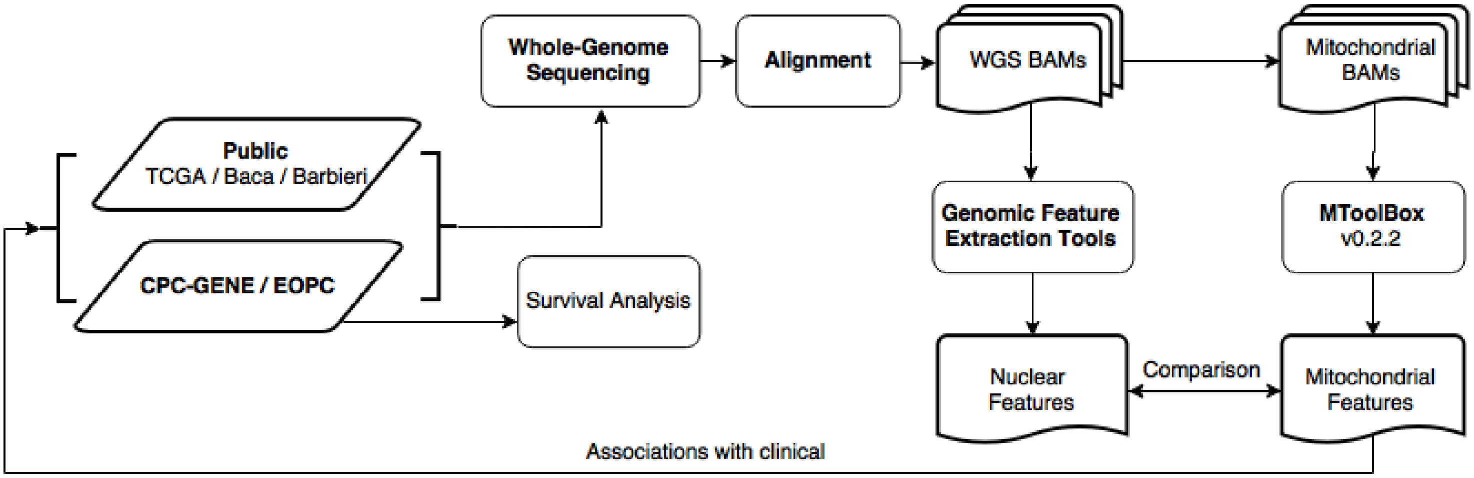

Supplementary Figure 1 | Experimental design. The experimental workflow for the project. Whole genome sequencing was performed on 333 CPCGENE and EOPC samples. In addition, 51 publicly available samples with whole genome sequences were included in the dataset and realigned. Mitochondrial reads were extracted and the mitochondrial analysis tool MToolBox was run on the resulting BAM files. Heteroplasmic fractions (HF) were calculated for each nucleotide and only those positions that differed by ≥ 0.2 HF between the tumour and matched normal were included in the list of somatic mutations.

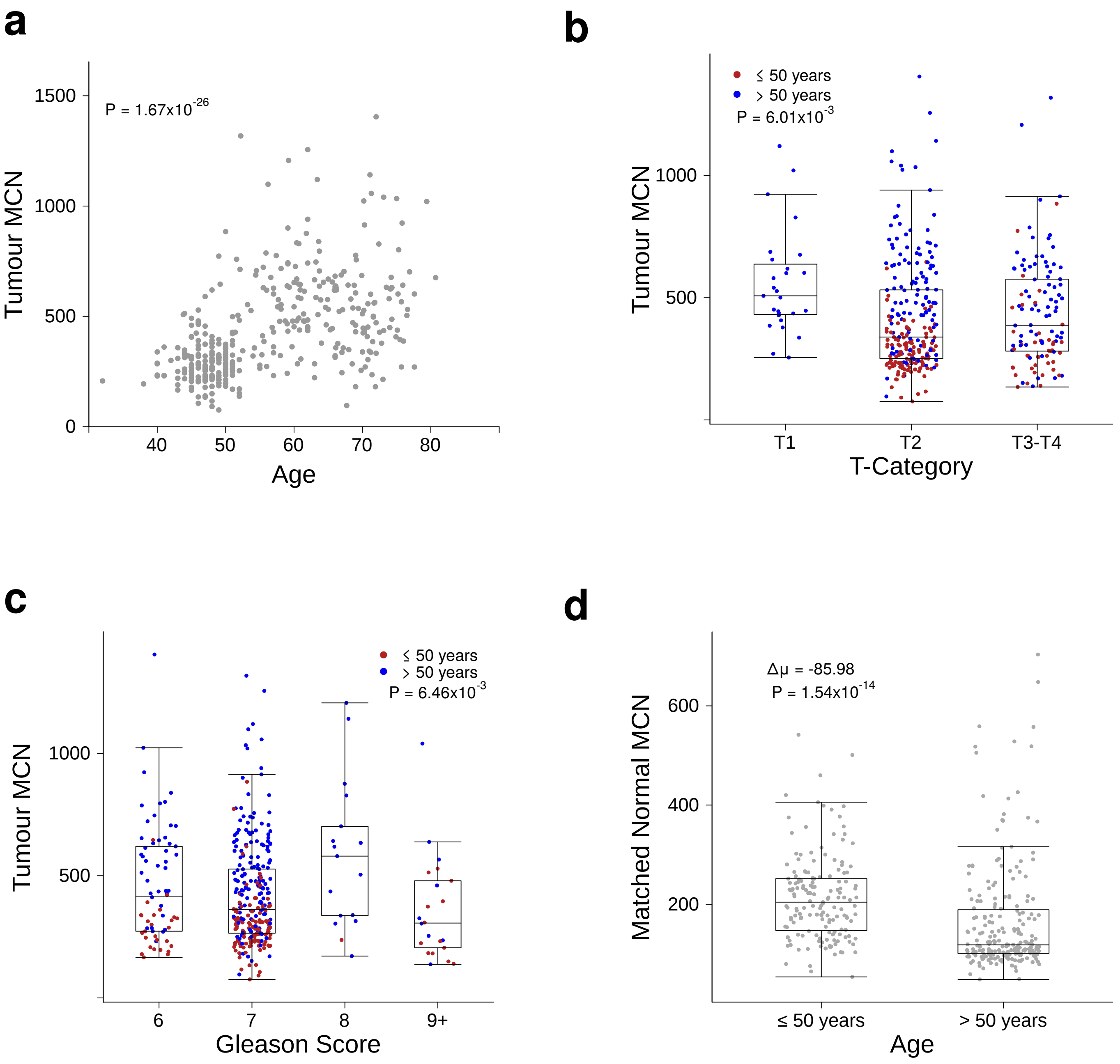

Supplementary Figure 2 | MCN association with clinical variables Tumour MCN categorized by age (**a**), T-category (**b**), and Gleason score (**c**). EOPC patients are indicated by red dots, LOPC by blue dots. (**d**) MCN of the matched normal samples show a significant difference between the two age groups.

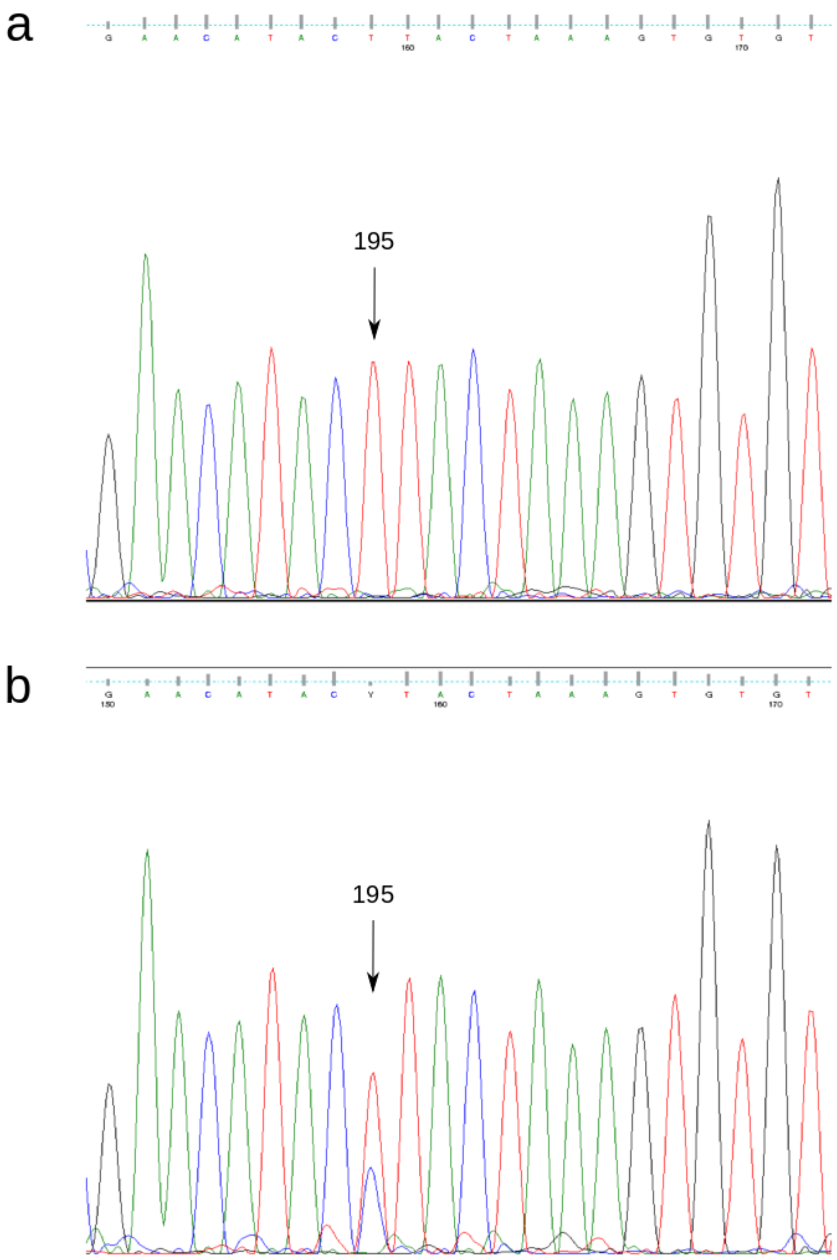

Supplementary Figure 3 | PCR validation confirms predicted mtSNVs. A comparison of chromatograms after PCR amplification and Sanger sequencing from (**a**) normal and (**b**) tumour samples from patient CPCG0196 for the mtDNA region: 187-208. Arrow indicates position 195 which has significant heteroplasmy in tumour.

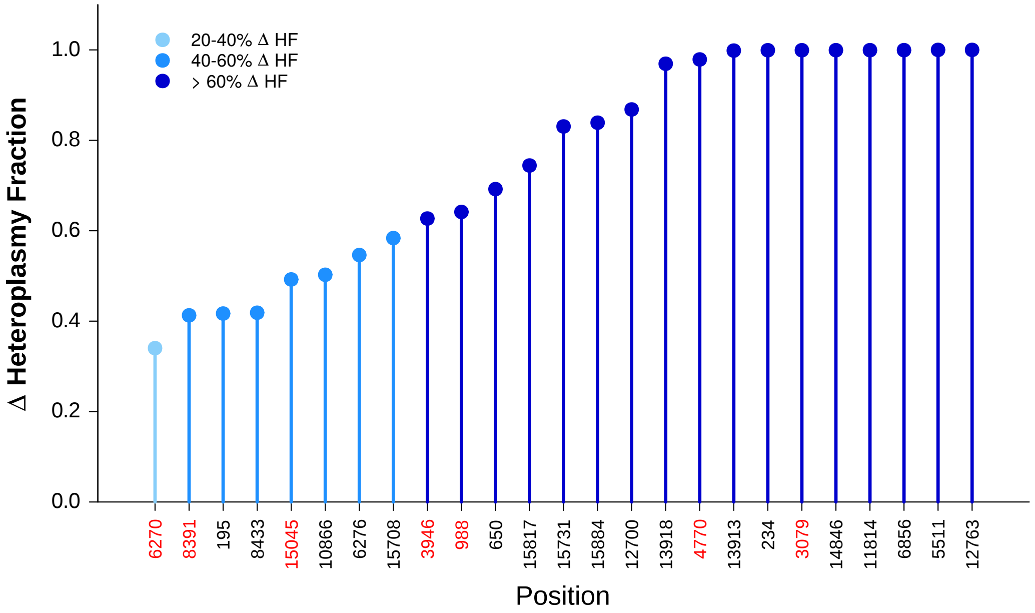

Supplementary Figure 4 | mtSNVs chosen for PCR validation. The 25 mtSNVs validated by PCR amplification and Sanger sequencing had varying levels in the difference in heteroplasmy (ΔHF) between tumour and normal samples. Light blue 20-40%, medium blue 40-60% and dark blue ≥60% ΔHF. mtDNA position on x-axis. Labels in red indicate those mtSNVs that failed PCR validation

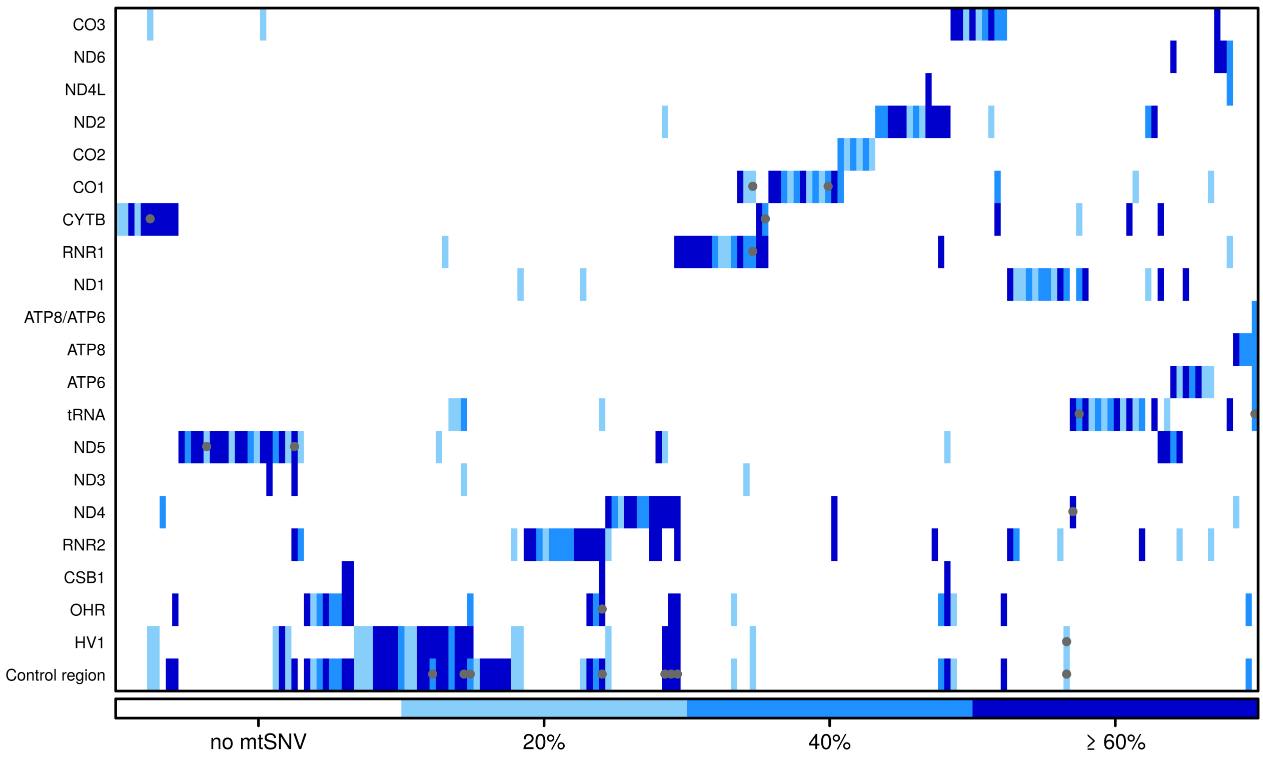

Supplementary Figure 5 | Frequency of mutations by patient and mitochondrial loci. Heatmap showing the distribution of mutations in the different mitochondrial regions (y-axis) by patients (x-axis). The difference in heteroplasmy fraction between tumour and normal sample (ΔHF) is indicated by colour, white: no mutation; light-blue: 20-40%; blue: 40-60% and dark blue ≥60%. Patients with more than one mtSNV in a particular mtDNA region are indicated by gray dots. Note: CSB1 and OHR are overlapping regions with the mtDNA Control region, mtSNVs in CSB1 are necessarily mtSNVS in OHR and both are mtSNVs within the Control region.

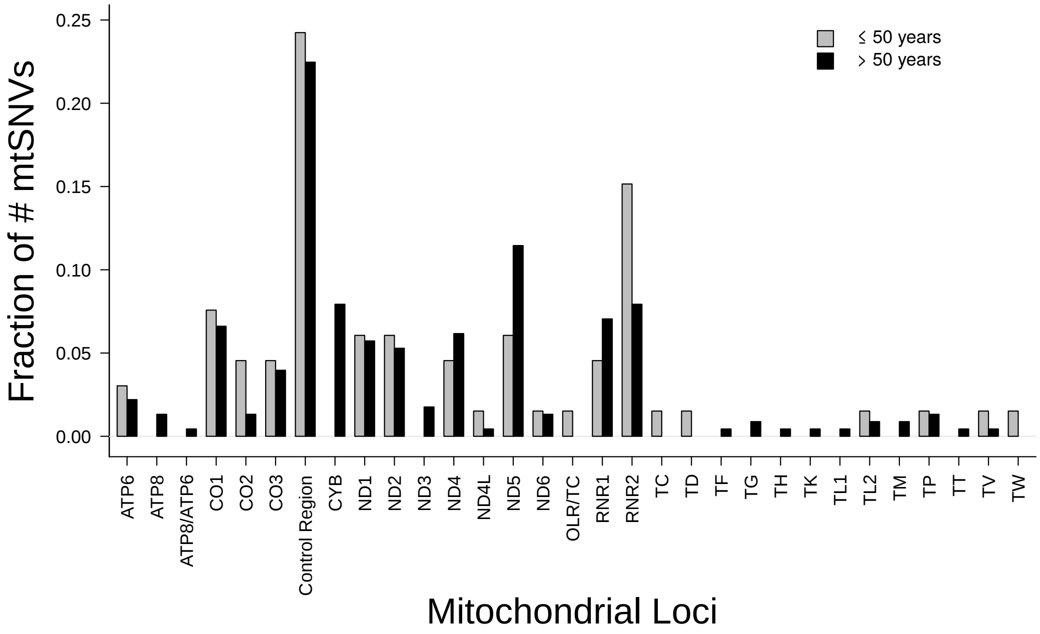

Supplementary Figure 6 | Distributions of mtSNV fractions by mitochondrial genome loci for EOPC and LOPC patients. The fraction of total mtSNVs per loci for EOPC and LOPC cohorts, those $\leq$ 50 years old (164 patients) and those > 50 year old (220 patients) respectively.

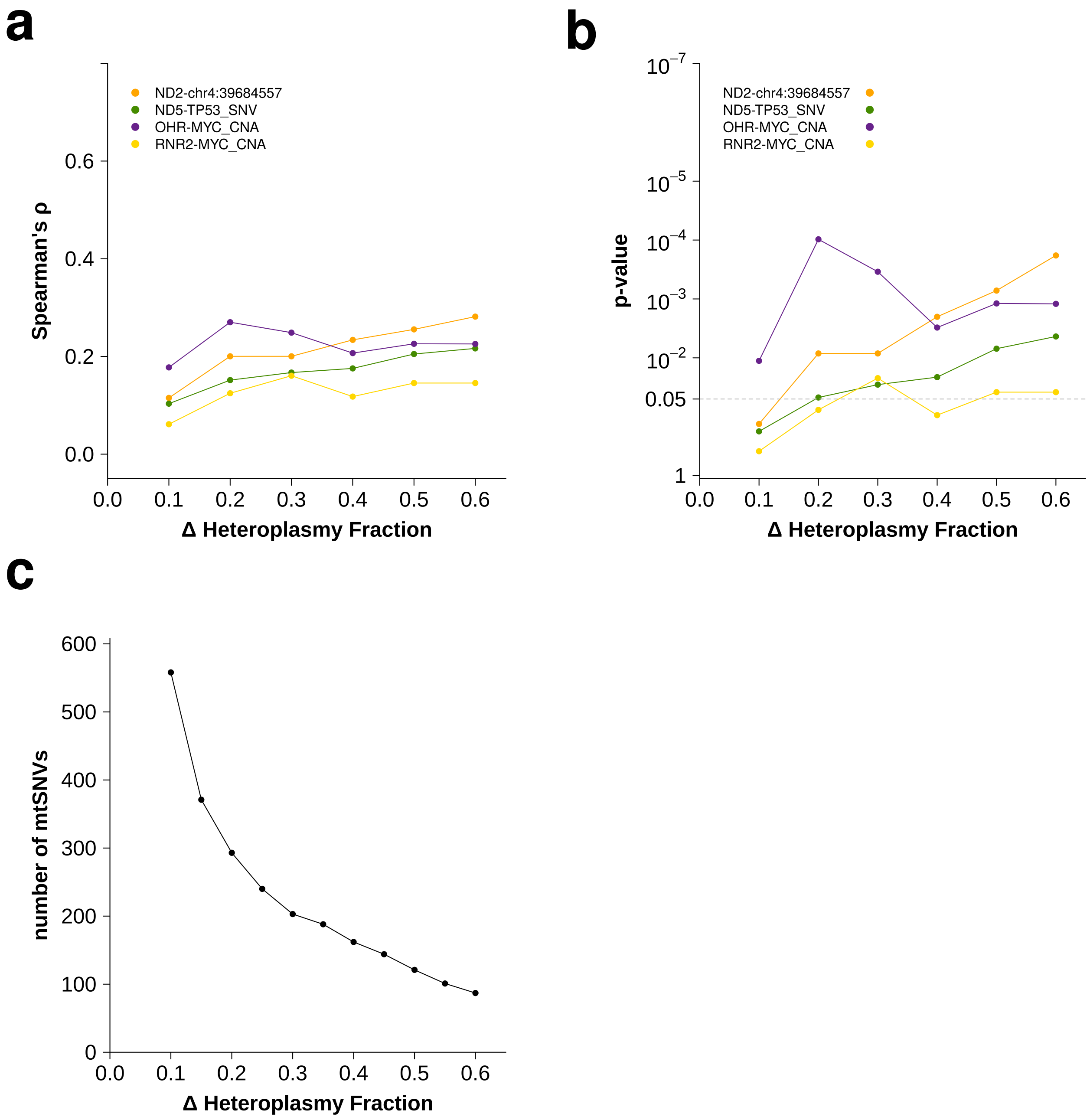

Supplementary Figure 7 | Correlations between nuclear and mitochondrial features as a function of heteroplasmy fraction. Spearman's ρ (**a**) and p-values (**b**) were calculated using increasing Δ HF cutoffs for mtSNVs for several nuclear and mitochondrial features: MYC CNAs and OHR mtSNVs, the non-coding SNV chr4:39684557 and ND2 mtSNVs, TP53 SNVs and ND5 mtSNVs, and MYC CNAs and RNR2 mtSNVs. (**c**) The total number of mtSNVs for 384 patients at each ΔHF threshold (unadjusted).

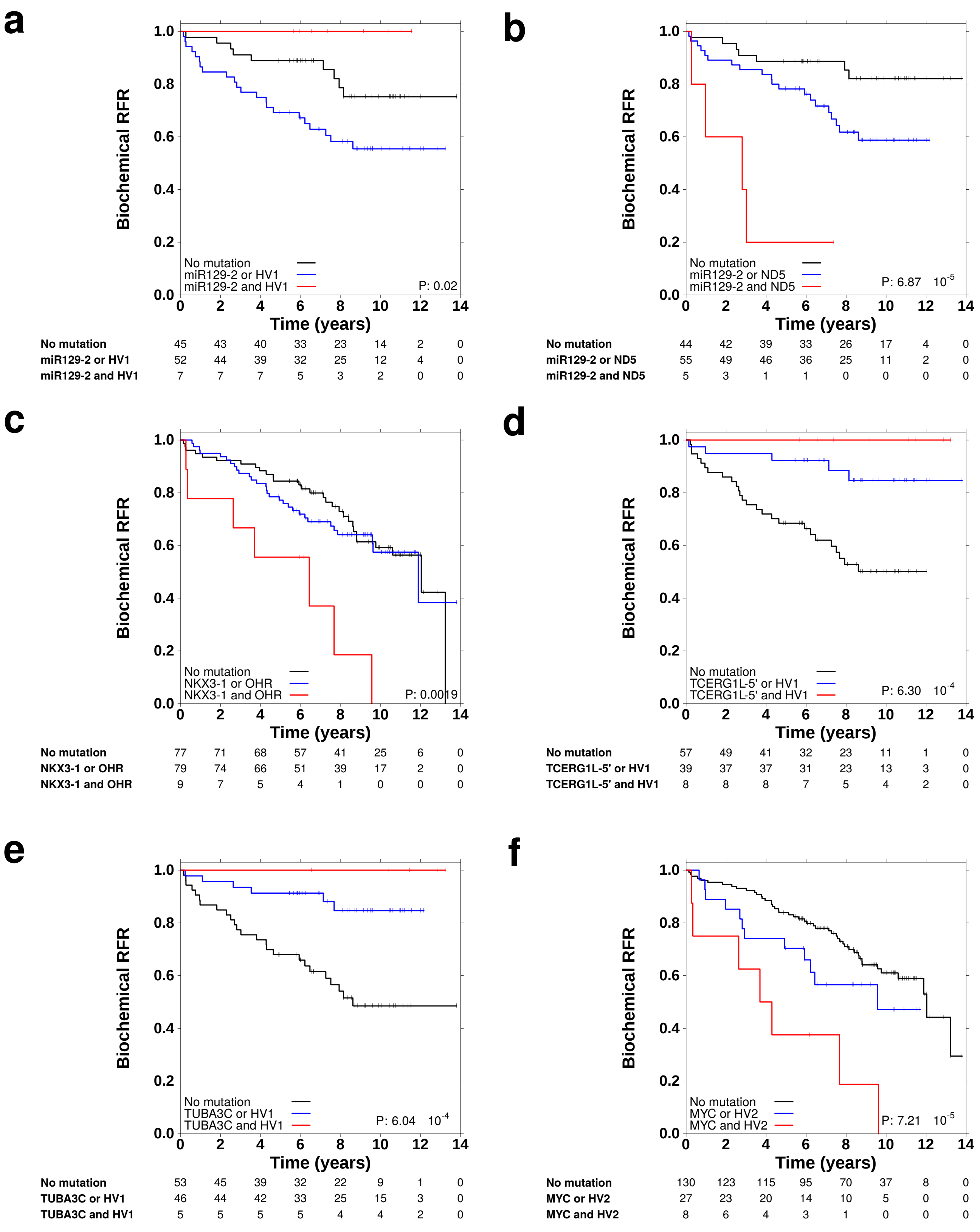

Supplementary Figure 8 | Prognostic synergy between mitochondrial and nuclear mutations. Kaplan-Meier plots of patients with (**a**) methylation events in miR129-2 and mtSNVs in HV1 or (**b**) ND5; (**c**) NKX3-1 CNAs and OHR mtSNVs; (**d**) mtSNVs in HV1 and methylation events in TCERG1L-5' or (**e**) TUBA3C; and (**f**) MYC CNAs and HV2 mtSNVs. Patients were grouped according to whether they had no mutations (black line), either a mtSNV or nuclear genomic mutation (blue) or had both (red line).

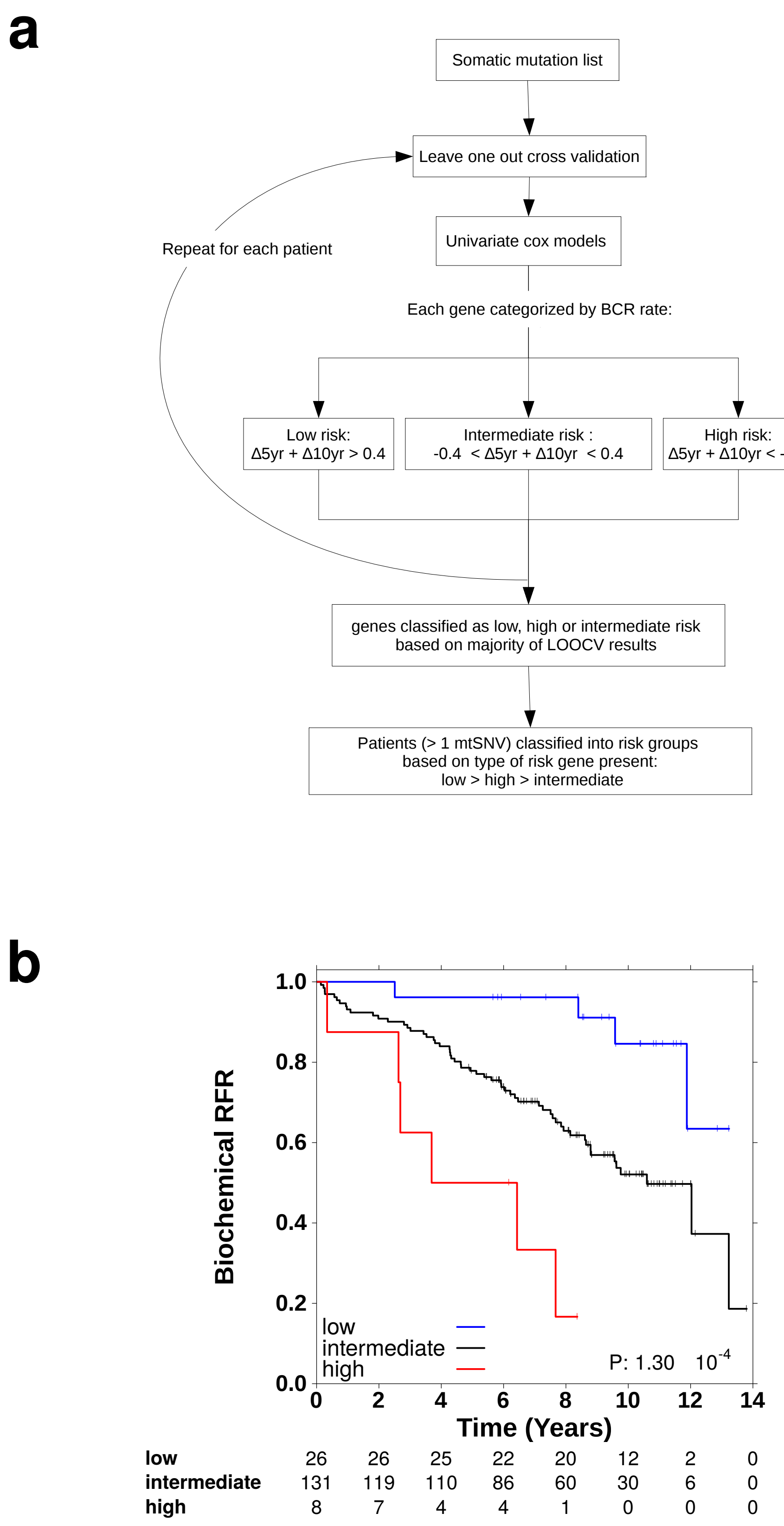

Supplementary Figure 9 | Experimental design for identifying mitochondrial signature. (**a**) Flowchart showing details of the leave-one-out cross validation method (**b**) Mitochondrial signature using a subset of three genes (HV1, OHR, CO3).

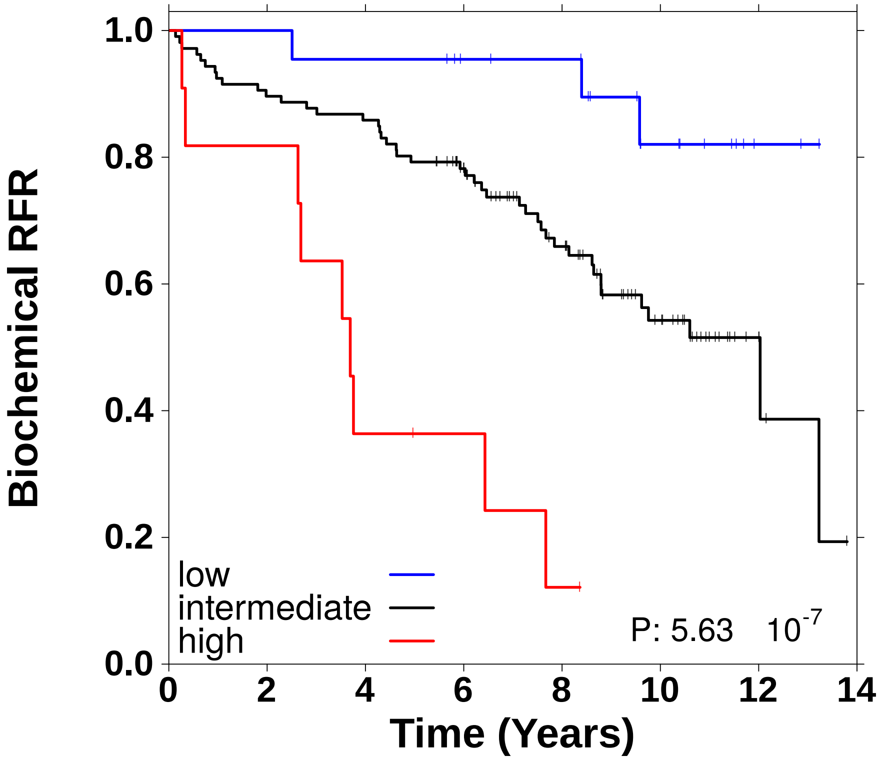

	0	2	4	6	8	10	12	14
**low**	22	22	21	18	17	9	2	0
**intermediate**	106	95	91	74	50	26	6	0
**high**	11	9	4	3	1	0	0	0

Supplementary Figure 10 | Mitochondrial signature in intermediate risk patients. Only patients classified as NCCN intermediate risk were used with the mtSNV signature and were separated into three risk-prediction groups, 'high' (red line), 'intermediate' (black line), and 'low' (blue line).

Supplementary Table 1: Results of PCR validation of 25 mtSNVs

Sample	Position	Amplicon	Validated?	HF - Tumou	HF - Normal
CPCG0196	195	1	Y	0.58T	1.0T
CPCG0242	234	1	Y	1.0G	1.0A
CPCG0189	650	2	Y	0.69C	1.0T
CPCG0248	988	3	N	0.64A	1.0G
CPCG0324	3079	5	N	1.0A	1.0G
CPCG0233	3946	6	N	0.63A	1.0G
CPCG0407	4770	7	N	0.98A	1.0G
CPCG0242	5511	9	Y	1.0C	1.0T
CPCG0189	6270	10	N	0.66G	1.0G
CPCG0251	6276	10	Y	1.0A	0.55G
CPCG0331	6856	11	Y	1.0C	1.0T
CPCG0345	8391	12	N	0.59G	1.0G
CPCG0196	8433	12	Y	0.58T	1.0T
CPCG0340	10866	14	Y	1.0T	0.5C
CPCG0410	11814	15	Y	1.0C	1.0T
CPCG0352	12700	16	Y	0.87A	1.0C
CPCG0236	12763	16	Y	1.0A	1.0G
CPCG0217	13913	17	Y	1.0C	1.0T
CPCG0410	13918	17	Y	1.0C	0.97T
CPCG0340	14846	18	Y	1.0A	1.0G
CPCG0412	15045	18	N	0.51G	1.0G
CPCG0412	15708	19	Y	0.58A	1.0G
CPCG0269	15731	19	Y	0.83A	1.0G
CPCG0269	15817	19	Y	0.91G	0.83A
CPCG0356	15884	19	Y	1.0A	0.84G

Supplementary Table 2: Results from univariate Cox proportional modeling

	Loci	HR	lower 95%	upper 95%	p-value (wald test)	p-value (logrank test)	10 year survival difference	number of patients with mitoSNV
1	Control Region	0.79	0.429	1.45	0.447	0.446	0.01	35
2	RNR1	1.48	0.671	3.28	0.329	0.326	-0.29	12
3	RNR2	1.25	0.538	2.91	0.603	0.602	-0.11	15
4	tRNAs	0.80	0.285	2.22	0.663	0.662	0.24	9
5	ND1	1.28	0.508	3.24	0.597	0.596	-0.03	10
6	ND2	1.27	0.507	3.16	0.613	0.613	-0.18	12
7	ND3	1.24	0.171	9.02	0.832	0.831	0.11	3
8	ND4	1.19	0.476	2.98	0.709	0.709	0.02	12
9	ND5	1.74	0.825	3.67	0.145	0.140	-0.24	16
10	ND6	1.69	0.234	12.25	0.601	0.597	-0.06	2
11	CO1	0.66	0.239	1.82	0.422	0.419	-0.02	12
12	CO2	0.00	0.000	Inf	0.997	0.196	0.45	3
13	CO3	0.25	0.035	1.80	0.168	0.136	0.35	9
14	ATP6	0.57	0.079	4.11	0.577	0.572	0.11	4
15	ATP8	4.25	1.014	17.84	0.048	0.031	-0.23	3
16	CYB	0.44	0.132	1.47	0.181	0.172	0.24	11
17	HV1	0.26	0.080	0.82	0.022	0.013	0.32	18
18	CSB1	4.52	1.410	14.46	0.011	0.005	-0.57	3
19	OHR	2.73	1.294	5.74	0.008	0.006	-0.41	12
20	MCN	1.50	0.918	2.46	0.105	0.103	-0.15	165
21	mtSNV	0.87	0.525	1.43	0.574	0.574	-0.047	105

Supplementary Table 3: PCR primers

Primer		Targets Region:
**1F**	tctatcaccctattaaccactcacg	10-385
**1R**	aggctggtgttagggttctttg	
**2F**	aaaaatttccaccaaacccccc	287-718
**2R**	actggaacggggatgcttg	
**3F**	acgggaaacagcagtgattaac	809-1103
**3R**	agggctaagcatagtggggt	
**4F**	cagcaaaccctgatgaaggc	1273-1710
**4R**	tggtagtaaggtggagtgggt	
**5F**	taccctagggataacagcgca	2927-3171
**5R**	gggaaggcgctttgtgaagt	
**6F**	tactcctgccatcatgaccct	3828-4067
**6R**	gttcaggggagagtgcgtc	
**7F**	ataccccgaaaatgttggttatac	4434-4927
**7R**	ggcttacgtttagtgagggagag	
**8F**	ctgacatccggcctgctt	4840-5171
**8R**	caggtgcgagatagtagtagggtc	
**9F**	catcgcccttaccacgctac	5457-5742
**9R**	cggcgggagaagtagattga	
**10F**	cataaacaacataagcttctgactcttacct	6192-6586
**10R**	ggtctcctcctccggcg	
**11F**	tatcctaccaggcttcggaat	6642-6957
**11R**	acctacggtgaaaagaaagatga	
**12F**	accacagtttcatgcccatc	8194-8482
**12R**	ggtgagggaggtaggtggtag	
**13F**	caaaaaggccttcgatacggg	9433-9876
**13R**	tagttggcggatgaagcagat	
**14F**	tgggcctagccctactagtctc	10688-10939
**14R**	gggtcggaggaaaaggttgg	
**15F**	tacgaacgcactcacagtcg	11760-11988
**15R**	tgtagagggagtatagggctgt	
**16F**	atctcgaactgacactgagcc	12524-12894
**16R**	aaggcgaggatgaaaccgat	
**17F**	ctaacaacatttcccccgcatc	13743-14056
**17R**	ggtggagatttggtgctgtg	
**18F**	cccaccccatccaacatctc	14811-15111
**18R**	caagcaggaggataatgccg	
**19F**	ggcgtccttgccctattact	15615-16051
**19R**	acccaaatctgcttccccat	
**20F**	atggggaagcagatttgggt	16032-16298
**20R**	gggtgggtaggtttgttggt	
